# The predictive value of bioimpedance-derived fluid parameters for cardiovascular events in patients undergoing hemodialysis

**DOI:** 10.1080/0886022X.2022.2095287

**Published:** 2022-07-20

**Authors:** Linghong Cheng, Liyang Chang, Rongrong Tian, Jianfang Zhou, Fenxia Luo, Hongmei Zhang

**Affiliations:** Department of Nephrology, Hangzhou TCM Hospital Affiliated to Zhejiang Chinese Medical University, Hangzhou, Zhejiang, China

**Keywords:** Bioimpedance spectroscopy, body composition monitor, fluid status, cardiovascular events, hemodialysis

## Abstract

**Background:**

It is becoming increasingly evident that the accurate assessment of fluid status is critical to ensure optimal care in patients undergoing hemodialysis (HD). Various fluid parameters, including overhydration (OH) and overhydration/extracellular water (OH/ECW%), which can be obtained using a bioimpedance spectroscopy device have been used to indicate the hydration status in such patients. This study aimed to explore the effect of these fluid parameters on cardiovascular events and determine which parameter was a better predictor of cardiovascular events (CVEs).

**Methods:**

A total of 227 patients who underwent HD at the Hangzhou Hospital of Traditional Chinese Medicine were enrolled in this prospective study between December 2017 and August 2018. Clinical data were collected, and the fluid status of patients was assessed using a body composition monitor. The patients were followed up until December 2020. The primary outcomes were CVEs. The association between fluid parameters and CVEs was analyzed using Cox proportional hazards models. The areas under the curve (AUCs) of receiver operating characteristic analysis and improvement in the global chi-squared value were used to compare the predictive values of fluid parameters for CVEs.

**Results:**

During a median follow-up of 31 months, 66 CVEs were recorded. The patients with a higher absolute hydration index (OH) and a relative hydration index (OH/ECW%) exhibited an increased risk of developing CVEs. After adjusting for confounding factors, both OH [hazard ratio (HR) 1.279 per L, 95% confidence interval (CI) 1.047–1.562; *p =* 0.016] and OH/ECW% (HR 1.061 per %, 95% CI 1.017–1.108; *p =* 0.006) were independently associated with CVEs. The predictive ability of the absolute hydration index was superior to the relative hydration index based on AUC calculations for CVEs. Furthermore, a greater change in *χ^2^* in predicting CVEs was noted for the absolute hydration index.

**Conclusions:**

Both absolute hydration index and relative hydration index were found to be independent predictors of CVEs in univariate and multivariate analyses. Furthermore, the absolute hydration index had a better additive predictive value than the relative hydration index in predicting CVEs.

## Introduction

Fluid overload is highly prevalent among patients undergoing hemodialysis (HD) and is strongly associated with poor outcomes, including systemic hypertension, pulmonary hypertension, left ventricular hypertrophy, and mortality [1]. Because patients may benefit from fluid overload management, it appears that the key first step is to accurately assess patient fluid volume status.

At most hemodialysis centers, the volume status is usually based on clinical criteria, including the patient’s signs and symptoms, blood pressure measurements, and intradialytic hemodynamic instability [2]. Although the presence of these signs is likely reflected in patient volume status, their lack of sensitivity and specificity is a major concern [3]. Therefore, objective tools are essential for assessing the hydration state. The bioimpedance spectroscopy (BIS) device is a non-invasive, economical, and highly reproducible method that rapidly provides information on the fluid status of patients undergoing HD [4]. More importantly, the BIS has been validated using isotope dilution methods [5,6], which are commonly considered the ‘gold standard’, although they are not routinely available. BIS device appears to be a promising and valuable tool for fluid management of patients undergoing HD [7–9]. Different bioimpedance-derived parameters have been used to define the volume status, including the absolute hydration index [overhydration (OH)] and the relative hydration index [OH/extracellular water (ECW)%]. Some previous studies have confirmed that fluid overload, detected using BIS, is useful for nephrologists to predict poor cardiovascular outcomes in patients with end-stage renal disease [10–12]. However, which parameter is the strongest predictor of cardiovascular events (CVEs) in patients undergoing HD is currently unknown. Therefore, this study aimed to elucidate the effect of different fluid parameters on CVEs and determine which parameter was the best predictor of CVEs in patients undergoing HD.

To the best of our knowledge, this study is the first to analyze and compare the predictive value of fluid parameters, measured using a portable whole-body bioimpedance spectroscopy device, for CVEs in a representative Asian cohort of patients undergoing HD.

## Materials and methods

### Study design and participants

This prospective study included patients from the HD Center of Hangzhou Traditional Chinese Medicine Hospital, People’s Republic of China, between December 2017 and August 2018. Patient inclusion criteria were as follows: aged between 18 and 80 years old; undergoing regular HD (three times a week in 4–4.5 h sessions). Patient exclusion criteria were as follows: those with clinical CVE during the 3-month period before enrolment; those with metallic joint prostheses, implanted defibrillators, or cardiac pacemakers; pregnant women, breastfeeding patients, or amputees; those with malignant tumors, severe liver disease, acute infections, or severe malnutrition. Finally, a total of 227 patients undergoing HD were recruited in the present study and followed up until December 2020. The study protocol was approved by the ethics committee of the Guangxing Hospital affiliated with the Zhejiang University of Traditional Chinese Medicine (No. 2018SQ119), and all the participants signed an informed consent form.

### Clinical characteristics

Demographic data, clinical history, etiology of renal failure, comorbidities, and dialysis data were collected from the patient's electronic medical records. We defined residual renal function as a 24-h urine output >200 mL [13]. Laboratory tests were performed using overnight fasting blood samples, which were obtained within 1 month of study enrolment. Dialysis efficiency was determined using Kt/V by a single-pool urea kinetic model.

### Bioimpedance spectroscopy analysis

The fluid status was assessed at baseline using a whole-body bioimpedance spectroscopy device (body composition monitor (BCM), Fresenius Medical Care, Bad Homburg, Germany) before dialysis. This technique involves attaching electrodes to the patient’s non-fistula forearm and ipsilateral ankle, with the patient in the supine position. All measurements were conducted by nursing staff trained in the manufacturer’s protocol. This device accurately measures body composition by analyzing electrical responses at frequencies between 5 and 1000 kHz. At low frequencies, the current cannot penetrate the cell membrane and instead passes through the ECW space, whereas at high frequencies, the current can flow through both ECW and intracellular water [14]. Based on a fluid model with these resistances, the ECW, intracellular water, total body water, and OH were calculated. Based on a three-compartment model, lean mass and fat mass were derived from the impedance data and were expressed as the lean tissue index and fat tissue index, respectively [15]. Volume status can be defined either as an absolute hydration index (OH) or as a relative variable, which reflects the excess ECW, and it can be calculated using the following formula:

Relative hydration index=OH/ECW×100%

#### Clinical outcomes

All patients were regularly followed up until 31 December 2020. In our study, the patients who followed up were treated with maintenance hemodialysis and came to the hospital for hemodialysis three times every week. Our investigation was therefore performed during this time. Outcome events were routinely registered in the system by experienced nephrologists. The endpoint events were CVEs, which included cardiac death, acute coronary syndrome, cerebrovascular accident, hospitalization for congestive heart failure, and acute peripheral artery occlusion [16]. In patients with more than one CVE, only the date of the first CVE was used in the subsequent statistical analysis. All patients were followed until the occurrence of CVEs, transfer to kidney transplantation, death, lost to follow-up, or December 2020.

#### Statistical analysis

Data were expressed as mean ± standard deviations for normally distributed data and as medians (interquartile range) for variables that did not follow a normal distribution and frequencies for categorical variables.

The Cox proportional hazards regression model was applied to perform univariate and multivariate analysis, which was presented as hazard ratios (HRs) and 95% confidence intervals (95% CIs). For multivariate Cox regression analysis, the enter method was applied. Baseline variables, include age, gender, body mass index (BMI), smoking status, presence of diabetes mellitus, previous CVEs, hemoglobin, serum albumin, highly sensitive C-reactive protein, serum calcium, serum phosphate, Kt/V, and total cholesterol, which could interfere with the association between fluid parameters and the endpoint were entered into multivariate Cox proportional hazards regression models. Given the number of events available, variables for inclusion were carefully chosen to ensure parsimony of the final models [17].

Receiver operating characteristic (ROC) curves were plotted and areas under the ROC curves (AUCs) were calculated to determine the discrimination threshold of each hydration value. Pairwise AUC comparisons were performed between fluid parameters using the DeLong test. The cut-off values appropriate for the optimal combination of sensitivity and specificity were determined using the Youden index. The incremental value of absolute hydration index and relative hydration index over the basic model was assessed to predict CVEs by assessing improvement in the global chi-squared value.

Kaplan–Meier curves were generated, showing cumulative probabilities of new CVEs, and differences were compared using the log-rank test.

These analyses were conducted using SPSS software, version 22.0 (IBM SPSS, Chicago, IL, USA) and MedCalc Statistical Software version 11.4.2.0 (MedCalc, Mariakerke, Belgium). All statistical tests were performed at a two-sided 0.05 level of significance.

## Results

### *Patient baseline charact*eristics *and end points*

**Figure 1.**
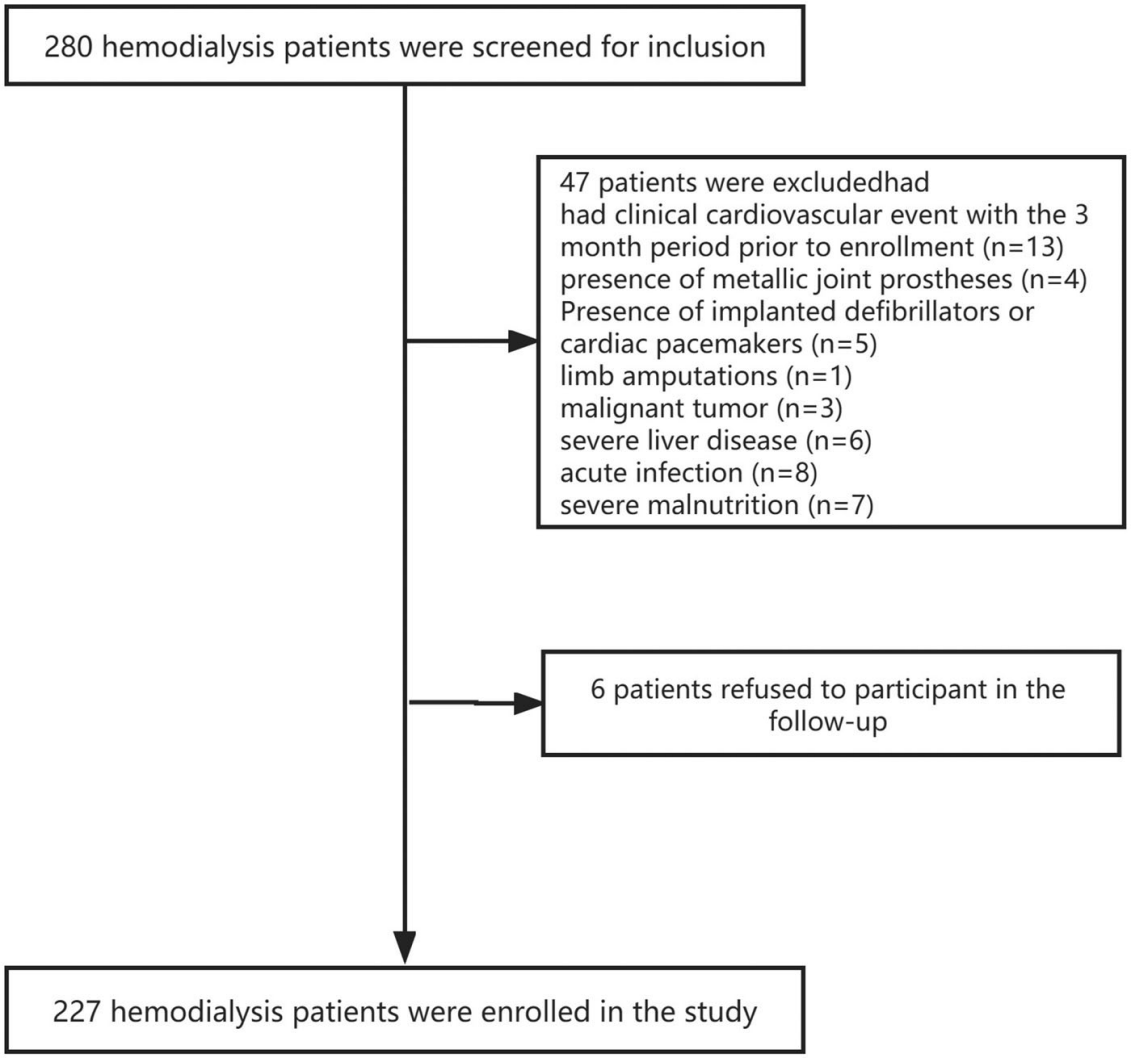
Flow diagram of participant selection and exclusion.

Figure 1 shows the flow chart of patients’ recruitment. Two hundred and twenty-seven patients undergoing HD were included in this study, with a mean age of 59.8 ± 12.8 years at baseline, a mean dialysis time of 71.7 ± 56.1 months, and a mean treatment time of 242.1 ± 7.7 min. Furthermore, 140 patients (61.7%) were men, 82 patients (36.1%) were diabetic, and 74 patients (32.6%) had a history of CVEs. The most common underlying kidney disease was glomerulonephritis (*n* = 131; 57.7%), followed by diabetic nephropathy (*n* = 56; 24.7%). At baseline, the mean absolute hydration index (OH) was 2.1 ± 1.4 L and the mean relative hydration index (OH/ECW%) was 13.6 ± 7.4%. Additional patient baseline characteristics of the cohort are shown in Table 1.

**Table 1.** Baseline characteristics of the study population.

Variable	All (*n* = 227)
Age (years)	59.8 ± 12.8
Male [*n* (%)]	140 (61.7%)
Residual kidney function, *n* (%)	61 (26.9%)
BMI (kg/m^2^)	21.4 ± 2.9
Ultrafiltration volume (L)	2.8 ± 0.9
Target weight (kg)	57.9 ± 9.7
Dialysis vintage (m)	71.7 ± 56.1
Treatment time per session (min)	242.1 ± 7.7
Current smoker [*n* (%)]	52 (22.9%)
Diabetes [*N* (%)]	82 (36.1%)
Hypertensive [*N* (%)]	175 (77.1%)
Previous CVEs [*N* (%)]	74 (32.6%)
Systolic BP (mmHg)	141 ± 22
Diastolic BP (mmHg)	79 ± 13
Cause of renal failure [*n* (%)]	
Glomerulonephritis	131 (57.7%)
Diabetic nephropathy	56 (24.7%)
Polycystic kidney	13 (5.7%)
Hypertensive nephrosclerosis	6 (2.6%)
Others/unknown	21 (9.3%)
Laboratory data	
Hb (g/L)	104.7 ± 11.6
hs-CRP (mg/L)	2.1 (0.9–5.8)
Alb (g/L)	38.5 ± 2.8
Ca (mmol/L)	2.3 ± 0.2
P (mmol/L)	1.8 ± 0.5
Scr (μmol/L)	884.3 ± 224.6
BUN (mmol/L)	24.1 ± 6.8
Kt/V	1.6 ± 0.3
TC (mmol/L)	4.2 ± 0.9
TG (mmol/L)	1.9 ± 1.3
Body composition measurements	
OH (L)	2.1 ± 1.4
OH/ECW	13.6 ± 7.4
LTI (kg/m^2^)	11.7 ± 2.4
FTI (kg/m^2^)	9.8 ± 3.5

BMI: body mass index; CVEs: cardiovascular events; BP: blood pressure; Hb: hemoglobin; hs-CRP: high sensitive C-reactive protein; Alb: albumin; Ca: serum calcium; P: serum phosphate; Scr: serum creatinine; BUN: blood urea nitrogen; TC: total cholesterol; TG: triglyceride; OH: Overhydration; OH/ECW: overhydration/extracellular; LTI: lean tissue index; FTI: fat tissue index.

Data are presented as means ± *SD*s or medians (interquartile ranges) for continuous variables and as *n* (%) for categorical variables.

In total, 66 CVEs and 23 all-cause deaths were recorded during a median follow-up of 31 months. For these 66 CVEs, the breakdown of the first events during follow-up were as follows: heart failure (*n* = 26), acute coronary syndrome (*n* = 19), cerebrovascular accident (*n* = 18), cardiac death (*n* = 2), and acute peripheral artery occlusion (*n* = 1).

### Comparing the predictive values of different indices of hydration

**Figure 2.**
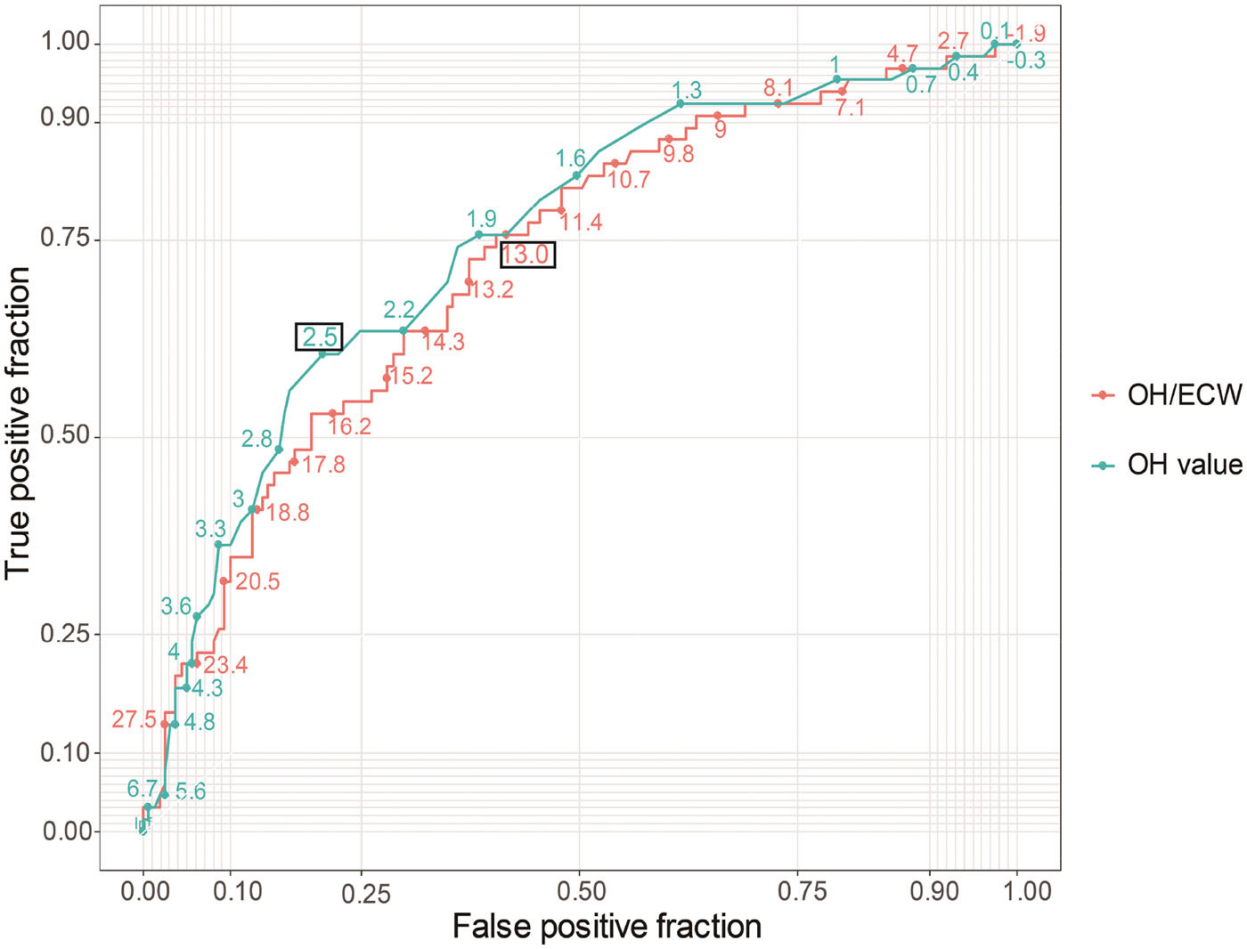
ROC curves of OH value and OH/ECW for prediction of cardiovascular events in HD patients.

The AUCs (Figure 2) for predicting CVEs are listed in Table 2. The AUCs of the OH and OH/ECW for predicting CVEs in patients undergoing HD was 0.750 (0.680–0.820) and 0.724 (0.653–0.796), respectively. Compared with the OH/ECW ratio, the performance of the OH value was superior in predicting CVEs (*z* *=* 2.413, *p* = 0.0158). According to the cut-off values of OH (2.5 L) and OH/ECW (13%), the sensitivity and specificity of OH for predicting CVEs in patients undergoing HD were 60.6 and 79.5%, respectively, and those of OH/ECW were 75.8 and 59.6%, respectively (Table 2). The details of sensitivity and specificity for different levels of absolute and relative hydration indices are shown in Supplemental Table 1.

**Table 2.** AUC uses ROC curve analyses to predict cardiovascular events.

	Cut point	Sensitivity	Specificity	AUC	95%CI	*p*-Value
Absolute hydration index (OH value)	2.5L	60.6%	79.5%	0.750	0.680–0.820	<0.001
Relative hydration index (OH/ECW)	13%	75.8%	59.6%	0.724	0.653–0.796	<0.001

ROC: receiver operating characteristic; AUC: area under curve; CI: confidence interval; OH: overhydration; OH/ECW: overhydration/extracellular.

**Figure 3.**
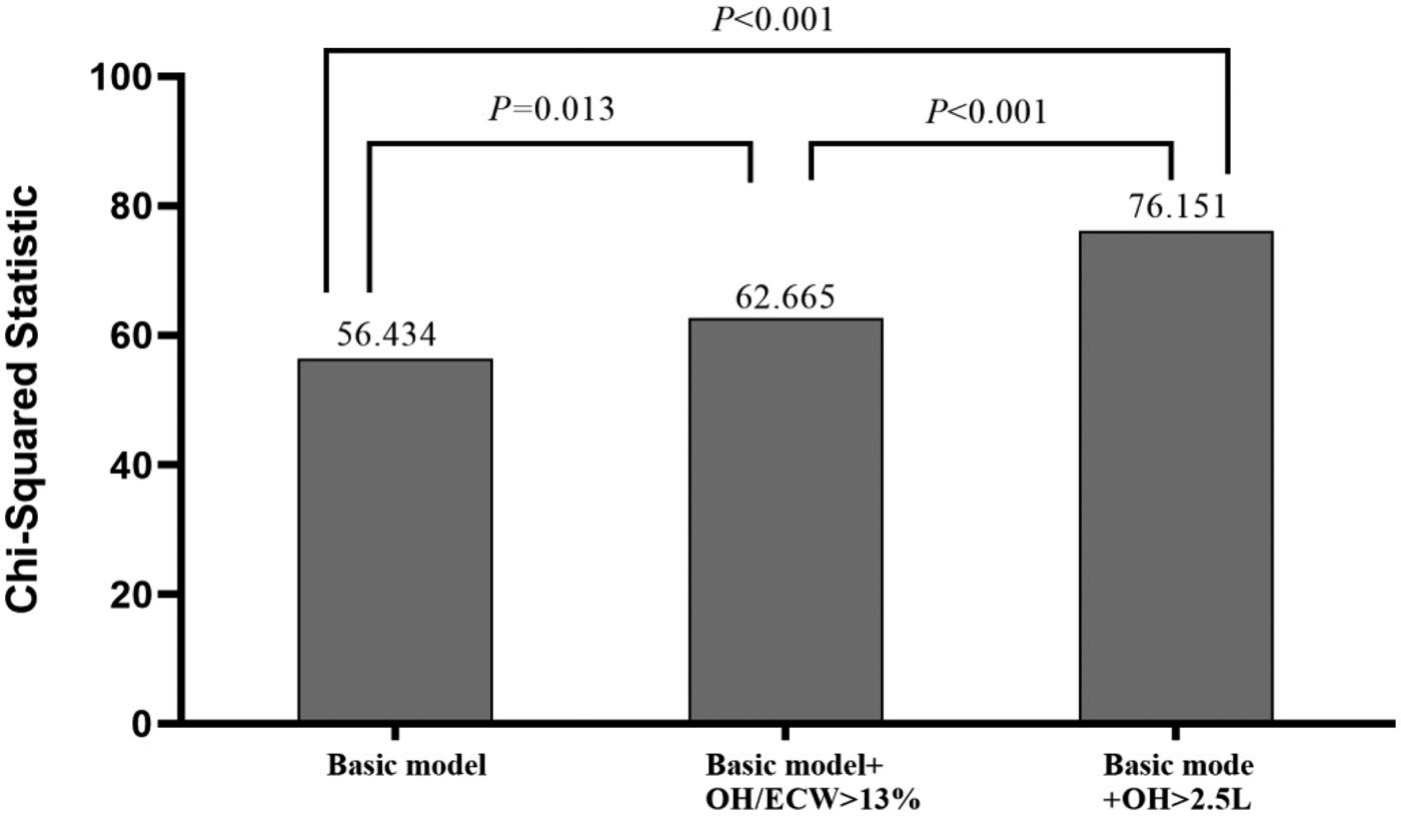
Comparison of the prediction power of the addition of fluid parameters to a basic model in the prediction of cardiovascular events.

The incremental values of fluid parameters used for predicting CVEs are shown in Figure 3. The basic model included the following parameters: age, gender, BMI, smoking status, presence of diabetes mellitus, and previous CVEs. The basic model could significantly predict CVEs (*χ^2^ =* 56.434, *p* < 0.001). We then added OH > 2.5 and OH/ECW > 13% into the basic model, and both basic model + OH >2.5 and basic model + OH/ECW >13% were more beneficial for the prediction of CVEs compared with the basic model (*χ^2^* change = 19.717, *p* < 0.001; *χ^2^* change = 6.231, *p =* 0.013, respectively). A direct comparison between the basic model + OH >2.5 and the basic model + OH/ECW >13% showed that the former had a better predictive value for CVEs (*χ^2^* change = 13.486, *p* < 0.001).

### Cardiovascular prognostic analysis

**Figure 4.**
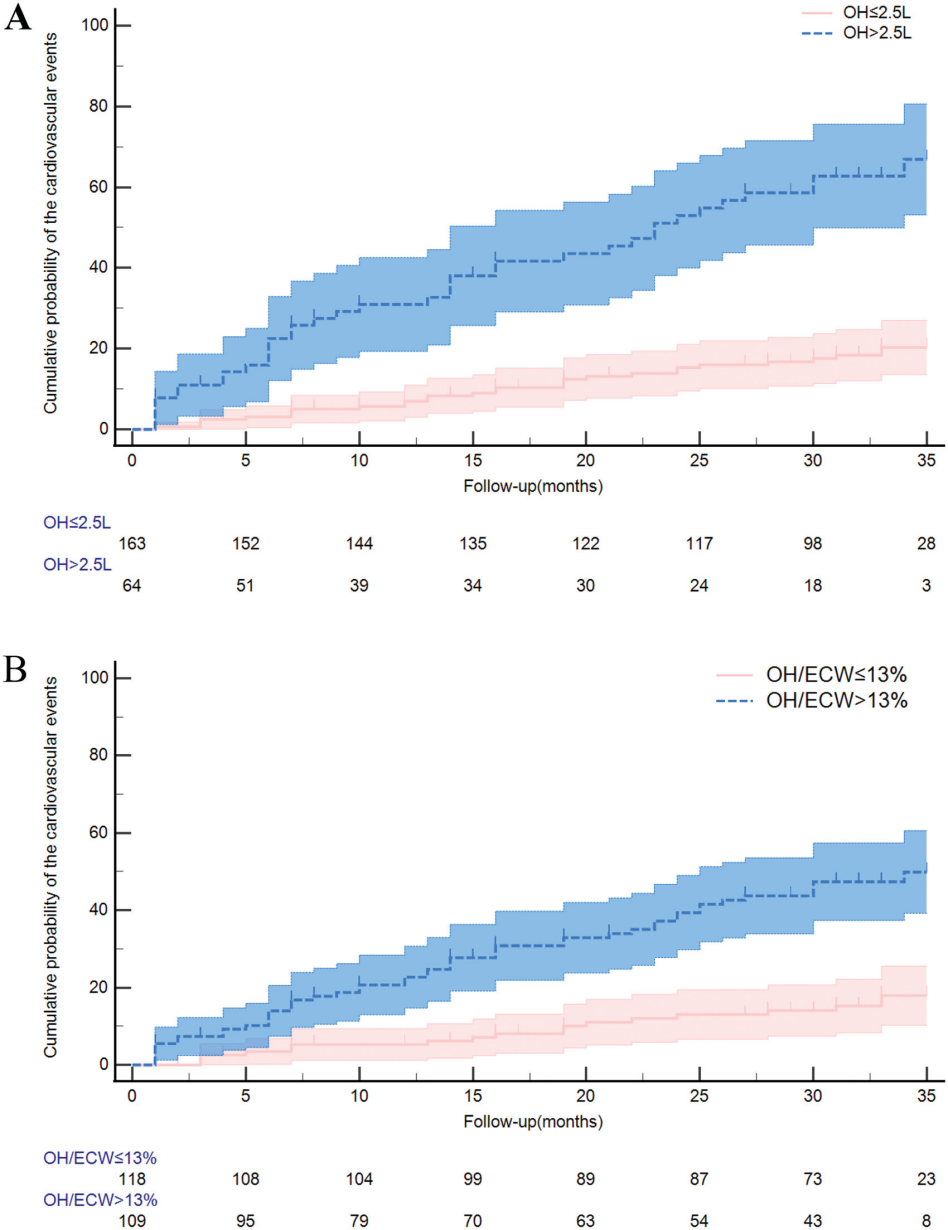
Kaplan–Meier analyses of cardiovascular events among (A) OH ≤ 2.5 L *vs.* OH > 2.5 L (log-rank *p* < 0.001); (B) OH/ECW ≤ 13% *vs.* OH/ECW > 13% (log-rank *p* < 0.001).

Figure 4 illustrates the Kaplan–Meier analyses of CVEs among the two study groups: (A) OH >2.5 L *vs.* OH ≤2.5 L (log-rank *p* < 0.001). (B) OH/ECW >13% *vs.* OH ≤13% (log-rank *p* < 0.001). A total of 109 (48%) patients had OH/ECW >13%, 64 (28%) patients had OH >2.5 L, and among the patients with OH >2.5 L, the OH/ECW ratio was all >13%. Patients with OH >2.5 L had a significantly higher risk of heart failure and acute coronary syndrome. Similar results were observed for OH/ECW >13% (Table 3). In the univariate analyses (Table 4), both the OH value and the OH/ECW ratio were found to be significantly associated with CVEs [HR = 1.515, 95% CI (1.330–1.725); *p* < 0.001 and HR = 1.100, 95% CI (1.066–1.136); *p* < 0.001, respectively]. After adjusting for various confounders, the multivariate Cox analyses (Table 5) revealed that the OH value and the OH/ECW ratio still remained independent risk predictors. Exemplarily, in the final adjusted model, the adjusted HR of CVEs with each 1 L increase in the OH value was 1.279 (95% CI 1.047–1.562; *p* = 0.016), and the adjusted HR of CVEs with each 1% increase in the OH/ECW ratio was 1.061 (95% CI 1.017–1.108; *p* = 0.006).

**Table 3.** Comparisons of clinical outcomes according to the cut-off OH value and OH/ECW ratio.

Variable	OH ≤ 2.5L (*n* = 163)	OH > 2.5L (*n* = 64)	*p*-Value	OH/ECW ≤ 13% (*n* = 118)	OH/ECW > 13% (*n* = 109)	*p*-Value
CVEs [*N* (%)]	29 (17.8%)	37 (57.8%)	<0.001	18 (15.3%)	48 (44.0%)	<0.001
Hospitalization for heart failure [*N* (%)]	10 (6.1%)	16 (25.0%)	<0.001	6 (5.1%)	20 (18.3%)	0.002
Acute coronary syndrome [*N* (%)]	8 (4.9%)	11 (17.2%)	0.003	4 (3.4%)	15 (13.8%)	0.005
Cerebrovascular accident [*N* (%)]	9 (5.5%)	9 (14.1%)	0.032	7 (5.9%)	11 (10.1%)	0.247

OH: overhydration; OH/ECW: overhydration/extracellular; CVE: cardiovascular event.

**Table 4.** Univariate Cox regression analysis for different factors as a risk for cardiovascular events.

Variable	HR	95%CI	*p*-Value
Parameter of hydration			
OH (per L)	1.515	1.330–1.725	<0.001
OH/ECW (per %)	1.100	1.066–1.136	<0.001
Age (per year)	1.044	1.021–1.067	<0.001
Gender (man *vs.* women)	2.902	1.607–5.242	<0.001
Residual kidney function (yes *vs.* no)	0.990	0.576–1.703	0.972
BMI (per 1kg/m^2^)	1.036	0.957–1.122	0.382
Ultrafiltration volume (per L)	1.233	0.935–1.625	0.137
Target weight (per kg)	1.032	1.007–1.057	0.012
Dialysis vintage (per month)	0.998	0.994–1.003	0.409
Treatment time (per min)	1.006	0.978–1.035	0.665
Current smoker (yes *vs.* no)	1.532	0.899–2.612	0.117
Diabetes (yes *vs.* no)	3.827	2.323–6.305	<0.001
Hypertensive (yes *vs.* no)	1.756	0.896–3.442	0.101
Previous CVEs(yes *vs.* no)	3.070	1.888–4.991	<0.001
Systolic BP (per mmHg)	1.011	0.999–1.022	0.063
Diastolic BP (per mmHg)	0.978	0.961–0.995	0.011
LTI (per kg/m^2^)	0.920	0.827–1.023	0.124
FTI (per kg/m^2^)	1.034	0.965–1.107	0.347
Laboratory data			
Hb (per g/L)	0.978	0.957–0.999	0.042
hs-CRP (per mg/L)	1.019	0.993–1.045	0.153
Alb (per g/L)	0.880	0.813–0.953	0.002
Ca (per mmol/L)	1.023	0.364–2.875	0.966
P (per mmol/L)	1.051	0.613–1.801	0.857
Scr (per μmol/L)	0.999	0.998–1.000	0.009
BUN (per mmol/L)	0.959	0.922–0.999	0.044
Kt/V (per increment of 1)	0.220	0.091–0.534	0.001
TC (per mmol/L)	0.699	0.526–0.929	0.014
TG (per mmol/L)	0.838	0.604–1.058	0.137

OH: overhydration; OH/ECW: overhydration/extracellular; BMI: body mass index; CVEs: cardiovascular events; BP: blood pressure; LTI: lean tissue index; FTI: fat tissue index; Hb: hemoglobin; hs-CRP: high sensitive C-reactive protein; Alb: albumin; Ca: serum calcium; P: serum phosphate; Scr: serum creatinine; BUN: blood urea nitrogen; TC: total cholesterol; TG: triglyceride.

**Table 5.** Multivariate Cox regression (enter method) analyses using different hydration parameters in each model.

Fluid parameters	HR (95%)	*p*-Value
OH value (per L)		
Model 1	1.525 (1.307, 1.779)	<0.001
Model 2	1.386 (1.160, 1.657)	<0.001
Model 3	1.279 (1.047, 1.562)	0.016
OH/ECW (per %)		
Model 1	1.093 (1.057, 1.131)	<0.001
Model 2	1.071 (1.031, 1.112)	<0.001
Model 3	1.061 (1.017, 1.108)	0.006

OH: overhydration; OH/ECW: overhydration/extracellular; HR: hazard ratio; CI: confidence interval. Model 1 adjusted for age, and gender. Model 2 adjusted for model I covariates and BMI, smoking status, presence of diabetes mellitus, and previous cardiovascular events. Model 3 adjusted for model 2 covariates and hemoglobin, serum albumin, high sensitive C-reactive protein, serum calcium, serum phosphate, Kt/V, and total cholesterol.

## Discussion

The present study evaluated the role of BCM fluid parameters in predicting the occurrence of CVEs in Chinese patients undergoing HD. Several major findings were noted in the present study. First, a high absolute hydration index and relative hydration index showed a significant correlation with an increased incidence of CVEs, which remained significant even after multivariable adjustment. Second, the optimal cut-off values of both absolute and relative hydration indices for predicting CVEs were 2.5 and 13%, respectively. Third, a direct comparison of three multivariate models revealed that the basic model + absolute hydration index > 2.5 had the highest predictive value for CVEs. To the best of our knowledge, this is the first study that analyzed and compared the predictive values of fluid parameters in a hemodialysis cohort with CVE end points.

BCM is a commercially available device that can provide information important in terms of fluid control [18]. The indices used in this study, which were herein assessed using BCM, namely the absolute hydration index and the relative hydration index, are easy to obtain and can be used to determine volume overload. Recently, Siriopol et al. [10] demonstrated that the absolute hydration index was associated with an increased risk of cardiovascular mortality. Furthermore, Tsai et al. [19] reported that higher OH/ECW was consistently associated with an increased risk for the combined endpoint of all-cause mortality and cardiovascular morbidity in patients with late-stage chronic kidney disease. In another cohort study, Onofriescu et al. [11] found that the relative hydration index was an independent predictor of CVEs. Our study provides evidence in favor of a deleterious effect of fluid parameters on CVEs in patients undergoing HD. Both high absolute and relative hydration indices were associated with increased incidence of CVEs even after adjusting for potential confounders. The absolute hydration and relative hydration indices are markers of fluid overload, which has an independent effect on vascular and endothelial levels, resulting in arterial stiffness, atherosclerosis, and left ventricular hypertrophy [12]. Furthermore, fluid overload can cause bowel wall edema with consequent translocation of endotoxins and bacteria [20]. These events, in turn, induce inflammatory processes [21–23], which are closely associated with malnutrition [24] and atherosclerosis [25], and increase the risk of CVEs. Bioimpedance-derived fluid parameters are readily available, non-invasive, and reproducible markers, it may be necessary for the physician to monitor regularly to prevent cardiovascular events in the early stage.

In addition, we constructed ROC curves to determine the optimal cut-off value for fluid parameters to predict CVEs. Our data showed that the cut-off values for OH and OH/ECW ratio in detecting CVEs were 2.5 L and 13%, respectively. In light of most literature, the 15% cut-off for OH/ECW ratio and 2.5 L for OH value was often defined as the upper limit of normohydration status, beyond which have been shown to predict poor prognosis [26–28]. Due to differences in research methods and study populations, the cut-off value of the OH:ECW ratio in our study was slightly lower than that in another research. It might represent new targets for the management of the HD population in Asia. Our analysis revealed that the OH/ECW cut-off value exhibited higher sensitivity (75.8 *vs*. 60.6%) but lower specificity (59.6 *vs*. 79.5%) for CVEs than those exhibited by the OH cut-off value. Therefore, the relative hydration index could be used as a better screening tool in terms of identifying high-risk patients. By contrast, the absolute hydration index expressed in liters could provide an estimate of how much the patient is overhydrated and have higher specificity in the detection of CVEs. Overall, the OH values displayed greater accuracy levels in the identification of CVEs than those displayed by OH/ECW. In our study, a direct comparison using multivariate models showed that the basic model + OH >2.5 L was superior to the basic model + OH/ECW >13% in predicting CVEs. Similar results were also obtained in the ROC comparison. Our study supports the use of the absolute hydration index to predict CVEs. This parameter is particularly useful because it can provide an estimate of how much the patient is overhydrated and allow the clinician to set a new target weight for the patient to achieve [29]. More importantly, by using a three-compartment model, the excess fluid is expressed as a separate compartment (OH), and the effects of age and gender were eliminated [30]. By contrast, OH/ECW may be affected by body composition. Lean tissue has a less relative proportion of ECW than adipose tissue. Therefore, since East Asians have relatively more lean tissue than Caucasians [31], they also have less relative ECW. Differences also exist between individuals, when a patient has an increased muscle mass, may have a reduced relative ECW, and this body composition effect could influence OH/ECW [32]. It seems that OH appears to be a better parameter for predicting CVEs than OH/ECW. However, further studies with a larger sample size are required to validate these results.

Our study had several limitations. First, the fluid status was defined using a single baseline measurement, and the relationship between the fluid status over time and outcomes could therefore not be analyzed. Second, additional confounding variables may have existed, which were not included in the multivariate-adjusted analysis. Third, our study had a relatively small sample size and was performed at a single center. Because of the small number of deaths, the influence of fluid parameters on mortality rates could not be further investigated.

In conclusion, our study revealed that both the absolute hydration index and the relative hydration index were independent predictors for CVEs in univariate and multivariate analyses. Besides, the absolute hydration index, which can be obtained non-invasively and economically, and without requiring additional calculations, showed a better predictive value than the relative hydration index in predicting CVEs. Our findings provide further evidence for the selection of the fluid index in predicting CVEs in patients undergoing HD. Future large, prospective trials are warranted to establish the benefits of BIA-guided fluid management in patients undergoing HD.

## Ethical approval

This study was reviewed and approved by the ethics committee of the Guangxing Hospital affiliated with the Zhejiang University of Traditional Chinese Medicine (No. 2018SQ119).

## Author contributions

Linghong Cheng analyzed the findings and drafted the manuscript. Liyang Chang and Rongrong Tian analyzed the data and revised the manuscript. Jianfang Zhou and Fenxia Luo revised the manuscript. Hongmei Zhang designed the study, revised the manuscript, and interpreted the results.

## Supplementary Material

Supplemental Material
