# Supplementary material for: The predictive value of bioimpedance-derived fluid parameters for cardiovascular events in patients undergoing hemodialysis
Source: Ren Fail. 2022 Jul 20;44(1):1192–200. doi: 10.1080/0886022X.2022.2095287 (PMC9318232; doi:10.1080/0886022X.2022.2095287)
Supplement: Supplemental Material [file IRNF_A_2095287_SM9698.pdf]

Supplemental Table1. Sensitivity and specificity for different levels of absolute and relative hydration index

	Sensitivity (%)	Specificity (%)
OH (L)		
1.0	92.4	26.7
1.5	83.3	50.3
2.0	69.7	65.2
2.5	60.6	79.5
OH/ECW (%)		
5	97.0	14.3
10	87.9	41.0
13	75.8	59.6
15	59.1	71.4

OH: Overhydration; OH/ECW: overhydration/extracellular.

Information Classification: General
